# Leverage structure and stock price synchronicity: Evidence from China

**DOI:** 10.1371/journal.pone.0235349

**Published:** 2020-07-01

**Authors:** Xiang Zhang, Han Zhou

**Affiliations:** 1 School of Economics, Chongqing Technology and Business University, Chongqing, China; 2 School of Finance, Nanjing University of Finance & Economics, Nanjing, China; The Bucharest University of Economic Studies, ROMANIA

## Abstract

This paper investigate the impact of leverage structure on stock price synchronicity. To better understand the mechanism of the impact of leverage, we break leverage into operating leverage and financing leverage. This breakdown reveals the impact of different component of leverage. Moreover, in this paper, we employ the quantile regression model to investigate the impact of leverage on different level of stock price synchronicity, which provides us a more comprehensive picture. Our empirical results show, operating and financing leverage have negative impact on stock price synchronicity. Moreover, the higher the stock price synchronicity becomes, the higher this impact is. Furthermore, the marginal effect of financing leverage will be attenuated as the profitability of firms becomes higher, but the interaction effect doesn’t exist for operating leverage. On the contrary, the marginal effect of financing leverage will be enhanced as the market capitalization of firms becomes larger, again, it doesn’t exist for operating leverage. Finally, as firms are state-owned, the marginal effect of both operating and financing leverage will be higher.

## 1 Introduction

Synchronicity incorporates market and industry level of information, lower synchronicity reflects more firm-specific information [1]. Recent research show that there is a negative correlation between price informativeness and stock price synchronicity. In other words, markets with lower synchronicity (higher idiosyncratic volatility) are more informative [2–4]. Hence, the study of the information content of synchronicity becomes very important. We join this literature by investigating the impact of leverage and its structure on stock price synchronicity.

Current research show that there is no consensus for the effect of leverage on stock price synchronicity. Generally, leverage is used as a control variable in econometric models, but the results differ in different research. First, findings show that the relationship of leverage and stock price synchronicity does not exist [5–7]. As considering the information technology innovation, in the subsample containing younger firms which are less than eight years, it is proved that higher leverage will lower idiosyncratic risk [8]. Opposite results claim that higher leverage incorporates more firm-specific information, then increases idiosyncratic risk [9–12].

This non-consensus may be caused by two reasons. First, in the studies of stock price synchronicity, two different measures of leverage are employed: The book value of total liabilities divided by total assets [5–7,11]. and the ratio of long-term (or short-term plus long-term) debt to total assets [8–10,12]. This difference causes the content of leverage differs, which leads to the different results. The second reason is that the mechanism of the impact of leverage has not been fully investigated. In this paper, we attempt to provide evidence of these two explanations. First of all, we break leverage into two parts: Operating leverage and financing leverage. Operating leverage arises when firms borrow money in running operations from customers and suppliers, and financing leverage occurs when firms borrow money to finance operations from investors [13]. We argue that, the effect of leverage is the combination of the two separate effects. Moreover, according to previous studies [13], the sum of operating leverage and financing leverage equals total liabilities divided by total assets. As a result, the first measure of leverage is employed in this paper. To prove the second reason, we investigate the interaction effects of leverage structure with other variables, because interaction effects reflect how the marginal effect of leverage structure is affected by other variables.

In this paper, with a sample of the Chinese stock market for the period from 2007 to 2016, our empirical results show that, total leverage is negatively correlated with stock price synchronicity, the same result is found for operating and financing leverage. These findings are consistent with current studies [9–12], which coincides with our expectation. Since leverage partially represents firm’s financial situation, which reveals corporate private information, as a result, firm’s stock price synchronicity decreases with leverage. Moreover, from the results of quantile regression, the impact of total leverage is higher when stock price synchronicity is lower, but on the contrary, the impact of operating leverage and financing leverage is higher when stock price synchronicity is higher. This result shows that the impact of capital structure is higher than leverage structure as the market becomes more informative. Furthermore, we investigate the interaction effects of leverage with three features of firms: profitability; market capitalization; state-owned feature. Our finding shows that, the marginal effect of leverage will be attenuated as the profitability of firms becomes higher, the same result is found for financing leverage, but the interaction effect doesn’t exist for operating leverage. On the contrary, the marginal effect of leverage will be enhanced as the market capitalization of firms becomes larger. Again, this interaction effect remains the same for financing leverage, but doesn’t exist for operating leverage. Finally, the marginal effect of leverage will be higher for state-owned firms, and this result is held for operating and financing leverage.

The reminder of this paper is organized as follows. The next section briefly reviews the literature. Measure of Variables and Model Specification are provided in section 3. Section 4 reports the data and analyzes the empirical results. Robustness check is represented in section 5. Section 6 concludes.

## 2 Literature review

### 2.1 Stock price synchronicity

The stock price movements are explained by systematic influences, industry influences, and firm-specific characteristics [1]. The measure *R*^2^ is called synchronicity which captures the changes in market and industry level. Thus, lower *R*^2^ incorporates more firm-specifics.

After the initial work of Roll, the growing literature focusing on synchronicity mainly consists of three aspects. First, the relationship between stock returns and synchronicity, which refers to idiosyncratic volatility puzzle. Previous studies [14] find that stocks with high idiosyncratic risk is associated with low average returns. This result violates the traditional asset pricing model’s prediction that they are either positively correlated or not correlated. Then many economists try to explain this puzzling result: After control of return reversal, the negative relation between realized idiosyncratic volatility and stock returns disappears, and the relation becomes positive when the conditional idiosyncratic volatility is estimated from EGARCH model [15,16]. It is also found that incomplete information partly explains the negative idiosyncratic risk premium [17]. Moreover, the idiosyncratic risk premium is positively correlated with stock returns on daily data, and negatively correlated with stock returns on monthly, quarterly and annual data by providing a GMM-type estimation procedure [18]. And some other explanations are found, for example: human capital [19], leverage [20], listing stock exchanges and liquidity [21, 22], corporate information disclosure [23], public news arrival [24] etc.

The second area of research consists in studying the power of explanation of synchronicity to other variables. Empirical evidence shows that where the synchronicity is lower, where the market is more informative [2–4, 23]. Moreover, the stock return synchronicity increases as market transparency (market informativeness) improves [10]. Hence stock price synchronicity can be proxied for market informativeness [12, 25–28]. In addition, idiosyncratic volatility is positively correlated to mispricing [29]. Idiosyncratic volatility can also be used as proxy for mispricing, and findings show that valuation uncertainty does not amplify the profitability premium in the Chinese stock market [30]. Meanwhile, idiosyncratic risk can also be used as the proxy for arbitrage cost, because arbitrageurs are unable to hedge this risk [31, 32].

The last part of studies tends to explain the information content of stock price synchronicity. Ownership structure plays an important role in stock price synchronicity. Initially, it is shown that institutional trading can accelerate the incorporation of firm-specific information [33]. However, it is also proved that stock price synchronicity is positively correlated to firm ownership by transient institutional investors, and this relation becomes negative to firm ownership by dedicated institutional investors, because transient investors tend toward arbitrage, and dedicated investors are inclined to monitor [6]. Evidence shows that synchronicity displays an inverted U-shape relation with the largest shareholder [5]. The effects of voting and cash flow rights are also investigated [7], the results show that stock price synchronicity increases with excess control and decreases when large shareholders own a large fraction of cash flow rights. Another explanation is analyst coverage. Evidence shows that stocks with more analyst coverage exhibit higher synchronicity [33, 34]. It is also proved that analysts produce more firm-specific information on stocks with higher idiosyncratic volatility [35]. Moreover, analyst coverage on firms with existing coverage conducts to decrease stock price synchronicity [36]. Financial reports quality also has a strong relation with stock price synchronicity. With a theoretical model, it is proved that opacity decreases idiosyncratic risk because it is shifted to managers [37, 38]. In addition, earnings quality is negatively correlated with idiosyncratic risk [39]. And stock price synchronicity increases with financial reports opacity [38]. Other explanations, for example: Firms with fewer antitakeover provisions lead to higher levels of idiosyncratic risk [9]. Industries adopting more intensively information technology exhibit higher firm-specific performance heterogeneity [8]. In terms of macroeconomic factors, product market competition can increase idiosyncratic risk [11]. it is also shown that there is an increasing trend for idiosyncratic volatility [40]. This trend can be explained by growth options [41].

### 2.2 Financing leverage and operating leverage

For the firms in the same risk class, a levered one should have a higher systematic risk than unlevered one [42, 43]. The idiosyncratic risk is defined as the dispersion of analysts’ forecasts and it is proved that, with an explicit theoretical model, for a levered firm, the expected returns decrease with idiosyncratic risk [20]. Current research show that the effect of leverage on stock price synchronicity is still ambiguous. Generally, leverage is used as a control variable in econometric models, but the results differ in different research. In addition, the measure of leverage is quite different as well. First, leverage is proxied by the book value of total liabilities divided by total assets. Findings show that the relationship does not exist when investigating the effect of largest shareholders [5–7]. When taking the production market competition into account, it is shown that a significant relation between leverage and stock price synchronicity, that is, a larger leverage increases idiosyncratic risk [11]. Another definition of leverage is the ratio of long-term (or short-term plus long-term) debt to total assets. The estimated results remain diverse. Previous findings are significant and consistent, higher leverage incorporates more firm-specific information, then increases idiosyncratic risk [9, 10, 12]. But when the information technology innovation is considered [8], the result is quite different. The coefficient of leverage is only significant in the subsample containing younger firms which are less than eight years. And the sign of coefficient is negative, which means that higher leverage will lower idiosyncratic risk.

Total leverage can be broken into operating and financing leverage. Operating leverage arises in running operations, and on the other hand, firms borrow money to finance operations, which creates financing leverage. It is documented that on average, the leverage effect of operating liability on profitability is greater than financing liability, because firms with profitable operating assets have more operating leverage and less financial leverage [44]. And the results are held in estimating future profitability. It is argued that profitability driven by operating activities has a stronger association with stock returns than the profitability driven by financing activities [45]. In addition, the operating/financing disaggregation can improve the forecasts of profitability when the components forecasting approach is used, but this disaggregation yields less accurate forecasts than the unusual/infrequent disaggregation [46].

Another classic definition of operating leverage is referred to as the ratio of the fixed to variable operating cost [47, 48]. They show that operating leverage has a positive relationship with the risk of stock price returns, and the degree of operating leverage can explain a large portion of the variation in market beta.

This paper focuses on the definition of operating/financing leverage which isolates the operating activities from the financing activities [49].

## 3 Variables and model specification

### 3.1 Stock price synchronicity

To measure stock price synchronicity, there are three ways in general. First, the standard deviation of residuals from Fama-French three-factor asset pricing model [50]. The second measure is also based on Fama-French three-factor regression, but an EGARCH model is applied in order to obtain the conditional idiosyncratic volatility. The third is classical method [1]. Because the first two measures are originated from asset pricing model, hence they are usually used in studying the relation between stock returns and idiosyncratic volatility. The last definition measures the firm-specific factors excluded from market and industry level. the third measure is employed in this paper because firm-specific volatility is the core variable studied.

We first estimate the following model:

$$r_{it}=\alpha +\beta_{1}{MKTRT}_{t}+\beta_{2}{MKTRT}_{t-1}+\beta_{3}{INDRT}_{t}+\beta_{4}{INDRT}_{t-1}+\varepsilon_{it}$$ (1)

where, *r*_*it*_ is the stock i’s return for time t, *MKTRT* and *INDRT* denote the market return and industry return respectively, and *ε* represents the unobserved random errors. All returns are daily based. The two common factors are value weighted.

Stock price synchronicity is defined as the variation of stock return explained by common factors, market based and industry based. This definition is equivalent to the *R*^2^ of the model (1). Since *R*^2^ is bounded in zero and one, then we apply a logistic transformation, hence the stock price synchronicity is defined as:

$${SYNCH}_{it}=\log\frac{R_{it}^{2}}{\left(1-R_{it}^{2}\right)}$$ (2)

where, *SYNCH*_*it*_ is the measure of synchronicity of stock *i* for year *t*.

### 3.2 Operating and financing leverage

In the specification of leverage [49], the operating liabilities arise from running businesses, such as accounts payable, advances from customers, etc. On the contrary, financing liabilities arise from raising fund to finance businesses, such as long-term debt, short-term borrowings, etc. In Table 1, a brief classification of operating and financing liabilities is represented.

**Table 1:** The classification of operating and financing liabilities.

Operating liabilities	Financing liabilities
Accounts payable	Short-term borrowing
Advances from customers	Long-term loans
Deferred income tax liabilities	Bonds payable
Accrued payroll	Long-term account payable
Tax payable	Accrued interest payable
Other liabilities	Current portion of non-current liabilities
Etc	Financial liabilities held for trading
	Etc

Because the sum of operating and financing liabilities is the total liabilities, then there is a simple way to deal with them. At the first step, we define financing liabilities, and then the difference between total liabilities and financing liabilities gives operating liabilities. In this paper, the following items are included in financing liabilities: short-term borrowing, long-term loans, bonds payable, long-term account payable, accrued interest payable, current portion of non-current liabilities, and financial liabilities held for trading. Then, operating liabilities equal to the difference between total liabilities and financing liabilities. In addition, instead of using the absolute value, we scale the two liabilities by total assets.

### 3.3 Model specification

To investigate the effect of leverage structure on stock price synchronicity, the following regressions are estimated:

$${SYNCH}_{it}=\alpha_{0}+\alpha_{1}{LEV}_{it}+\sum_{k}\alpha_{k}{CONTROL}_{it}^{k}+\varepsilon_{it}$$ (3)

$${SYNCH}_{it}=\beta_{0}+\beta_{1}{OPDT}_{it}+\beta_{2}{FINDT}_{it}+\sum_{k}\beta_{k}{CONTROL}_{it}^{k}+\varepsilon_{it}$$ (4)

where, for firm *i* and year *t*, *LEV* stands for leverage, defined as book value of total liabilities divided by total assets. *OPDT* represents the ratio of firm’s operating liabilities to total assets, *FINDT* equals to the financing liabilities to total assets. *CONTROL* denotes a set of control variables. *ε* represents the unobserved random errors.

Eight control variables are included in this paper. Institutional shareholder (INST) is included, and defined as the percentage of shares holding by institutional investors, because institutional trading can accelerate the incorporation of firm-specific information [6, 33]. We also include liquidity proxied by turnover (TURN), which is computed as the average of daily turnover during the year, the volatility caused by firm-specific information increases with liquidity [51]. Return on assets (ROA) is added as well, because ROA is one of firm’s fundamentals. As a result, firms with higher ROA exhibit larger impact on stock price synchronicity. We add firm size (SALE) as a control variable, which is defined as the natural logarithm of total sales at the end of the year. We set a dummy variable STATE which is equal to 1 if the firm is state-owned. In China, there are a number of state-controlled companies, their objective is not just profit maximization, but also social and political intention, as a result, state-owned companies are with less firm-specific information [52]. Finally, Tobin’s q (TOBIN) is added as one of firm’s specifics. In addition, we include two macroeconomic factors: the economic growth and monetary policy, the former is defined as the economic growth rate (GDP) and the latter is defined as the annual mean of one-week Shanghai Interbank Offering Rate (SHIBOR).

## 4 Data and empirical results

### 4.1 Data

Our sample contains the annual data from 2007 to 2016. The stock return and accounting data are extracted from the China Stock Market and Accounting Research (CSMAR) database, and the institutional ownership data is extracted from WIND database. Stock return data are originally daily data, and daily market return and industry return are computed in a value-weighted way, and then the stock price synchronicity is obtained by running the regression of Eq (1) for each fiscal year. Table 2 shows the detailed definition of all variables. We exclude the outliers if they are outside of twice the interquartile range. Our initial sample contains 2,997 firms and 21,946 observations, after the elimination of outliers, the final sample size is reduced to 2,818 firms and 18,707 observations.

**Table 2:** This table represents definition of variables.

Variable	Definition
**SYNCH**	Stock price synchronicity estimated from Eq (2)
**OPDT**	Operating liabilities divided by total assets
**FINDT**	Financing liabilities divided by total assets
**LEV**	Total liabilities divided by total assets
**ROA**	Return on assets of firms for each year
**SALE**	Natural logarithm of total sales at the end of each year
**SIZE**	Natural logarithm of market capitalization at the end of each year
**STATE**	Dummy variable for state-owned company
**INST**	Percentage of shares held by institutional investors
**TURN**	Annual mean of daily turnover
**TOBIN**	Tobin’s Q at the end of each year
**GDP**	Annual growth rate of GDP based on year 2007
**SHIBOR**	Annual mean of 7-days SHIBOR

Table 3 reports the descriptive statistics of variables. All variables are positively skewed except for SYNCH and SHIBOR. INST contains many missing observations due to data collection problem. Table 4 reports the correlations of variables, which shows no multicollinearity between variables.

**Table 3:** This table reports the summary information for all variables.

Variables	N	Mean	S.D	Skewness	Kurtosis	P25	P50	P75	Min	Max
SYNCH	18695	-1.030	0.930	-0.310	4.660	-1.570	-1.060	-0.410	-9.120	5.650
OPDT	18705	0.240	0.140	0.840	3.270	0.140	0.210	0.320	-0.0200	0.720
FINDT	18705	0.210	0.170	0.620	2.700	0.0500	0.180	0.330	0	0.860
LEV	18705	0.450	0.210	0.0100	2.210	0.290	0.450	0.610	0.0100	1.200
ROA	18705	0.0400	0.0400	0.190	3.950	0.0100	0.0400	0.0600	-0.0900	0.170
SALE	18705	21.32	1.440	0.360	4.010	20.37	21.20	22.14	12.69	28
STATE	18707	0.460	0.500	0.150	1.020	0	0	1	0	1
INST	18368	0.370	0.230	0.180	2.030	0.170	0.360	0.550	0	0.990
TURN	18706	0.0300	0.0200	1.110	3.850	0.0100	0.0200	0.0400	0	0.110
TOBIN	18705	1.890	0.830	1.230	4.020	1.270	1.650	2.300	0	4.740
GDP	18707	3.590	0.770	1.400	4.670	3.060	3.300	3.940	2.820	5.770
SHIBOR	18707	3.040	0.810	-0.490	2.510	2.370	2.920	3.580	1.230	4.080

**Table 4:** This table shows the correlations of variables.

	SYNCH	OPDT	FINDT	LEV	ROA	SIZE	STATE	INST	TURN	TOBIN	GDP	SHIBOR
SYNCH	1											
OPDT	0.022***	1										
FINDT	0.058***	-0.098***	1									
LEV	0.062***	0.589***	0.747***	1								
ROA	-0.079***	-0.116***	-0.399***	-0.402***	1							
SALE	0.161***	0.346***	0.295***	0.471***	0.017**	1						
STATE	0.144***	0.197***	0.222***	0.312***	-0.148***	0.235***	1					
INST	0.102***	0.154***	0.123***	0.203***	0.074***	0.543***	0.280***	1				
TURN	-0.051***	-0.122***	-0.147***	-0.201***	0.017**	-0.312***	-0.237***	-0.503***	1			
TOBIN	-0.023***	-0.078***	-0.279***	-0.279***	0.195***	0.170***	-0.169***	0.050***	0.188***	1		
GDP	-0.257***	0.028***	0.084***	0.087***	0.068***	-0.263***	0.162***	-0.181***	0.156***	-0.043***	1	
SHIBOR	-0.292***	-0.052***	-0.044***	-0.070***	-0.00600	-0.137***	-0.082***	0.042***	-0.272***	-0.198***	-0.191***	1

*** indicates significance at the 1% level

** indicates significance at the 5% level, and

* indicates significance at the 10% level.

### 4.2 Impact of leverage structure on stock price synchronicity

Empirical results are shown in Table 5. Results show that LEV, OPDT and FINDT are negatively correlated with SYNCH. This is quite obvious, because leverage is part of firm’s fundamentals, which stands for the private information of firms, then, firm’s leverage will lower stock price synchronicity. Moreover, the impact of FINDT is slightly larger than OPDT. For control variables, ROA, SIZE, TURN and TOBIN also have negative impact on stock price synchronicity for the same reason, they are also firm-specifics. The coefficient is positive for STATE, which means that, state-owned companies help to incorporate market-level and industry-level information. The coefficient of INST is positive, which is consistent with previous studies [6]. The effect of two macroeconomic factors differ; when individual and time fixed effect are controlled, the increase of economic growth helps to incorporate more private information, but the monetary policy has an opposite effect.

**Table 5:** Results of estimation of the impact of leverage structure on stock price synchronicity. Dependent variable: SYNCH. Two different estimation methods are used. Columns (1) and (2) represent pooled regression with White-robust standard errors; We control the individual and time fixed effect with White-robust standard errors in columns (3) and (4).

	(1)	(2)	(3)	(4)
LEV	-0.225***		-0.248***	
	(0.0357)		(0.0613)	
OPDT		-0.283***		-0.232***
		(0.0455)		(0.0863)
FINDT		-0.176***		-0.258***
		(0.0430)		(0.0708)
ROA	-1.104***	-1.043***	-0.927***	-0.935***
	(0.177)	(0.180)	(0.201)	(0.203)
SALE	0.117***	0.115***	-0.157***	-0.156***
	(0.00753)	(0.00757)	(0.0179)	(0.0179)
STATE	0.243***	0.245***	0.117***	0.117***
	(0.0135)	(0.0135)	(0.0415)	(0.0415)
INST	-0.200***	-0.198***	0.0596	0.0597
	(0.0358)	(0.0358)	(0.0468)	(0.0468)
TURN	-1.750***	-1.751***	-4.556***	-4.555***
	(0.393)	(0.393)	(0.468)	(0.468)
TOBIN	-0.114***	-0.112***	-0.0718***	-0.0721***
	(0.00837)	(0.00842)	(0.0119)	(0.0120)
GDP	-0.375***	-0.376***	-0.565***	-0.564***
	(0.00826)	(0.00827)	(0.0105)	(0.0105)
SHIBOR	-0.408***	-0.409***	0.246***	0.246***
	(0.00763)	(0.00762)	(0.0510)	(0.0510)
CONS	0.176	0.199	3.693***	3.688***
	(0.128)	(0.129)	(0.301)	(0.301)
Firm FE	No	No	Yes	Yes
Year FE	No	No	Yes	Yes
R-sq	0.235	0.235	0.477	0.477

*** indicates significance at the 1% level, ** indicates significance at the 5% level, and * indicates significance at the 10% level. Standard errors are reported in parentheses.

### 4.3 Impact of leverage structure on different level of stock price synchronicity

Table 5 shows us the results of mean regression of the impact of leverage structure on stock price synchronicity, which is that, on average, the stock price synchronicity decreases with leverage structure. But it is also very important to understand the effect of leverage structure at different level of stock price synchronicity. The quantile regression provides a more complete information. In this section, we use the quantile regression to investigate the asymmetric effect. We estimate seven quantiles, from the lower (0.05) to the higher one (0.95).

The empirical results are reported in Table 6. To save space, the estimation of results for other control variables are not reported. The results show that the impact of leverage structure on different level of stock price synchronicity differs. The impact of LEV is higher when stock price synchronicity is lower and it tends to decrease as stock price synchronicity increases, which means that, when stock market is more informative, more information of leverage will be incorporated in stock prices. But the impact of OPDT and FINDT fluctuates as stock price synchronicity changes. Moreover, on the contrary, the impact of OPDT and FINDT is higher when stock price synchronicity is higher, and this effect is lower while stock price synchronicity is lower. As a result, as stock market is less informative, more information of OPDT and FINDT will be revealed; when stock market is more informative, less information of OPDT and FINDT will be revealed. This result shows that, as stock market becomes more informative, the impact of capital structure will be higher than leverage structure.

**Table 6:** Results of estimation of the impact of leverage structure on stock price synchronicity with quantile regression. Dependent variable: SYNCH. We estimate a quantile regression model for panel data (QREGPD) with nonadditive fixed effects. Adaptive MCMC optimization technique is applied, 1000 draws are performed, and 100 draws to drop as a burn-in period. Acceptance rate is set to 0.5. To save space, the estimation of results for other control variables are not reported.

	q05	q10	q25	q50	q75	q90	q95
LEV	0.0933**	-0.139***	-0.352***	-0.387***	-0.328***	-0.359***	-0.300***
	(0.0383)	(0.0156)	(0.0240)	(0.0101)	(0.0109)	(0.0121)	(0.0141)
OPDT	-0.280***	-0.195***	-0.314***	-0.452***	-0.317***	-0.348***	-0.472***
	(0.0586)	(0.0407)	(0.0159)	(0.0676)	(0.0474)	(0.0176)	(0.0180)
FINDT	-0.195***	-0.189***	-0.158***	-0.344***	-0.328***	-0.171***	-0.348***
	(0.0750)	(0.0310)	(0.0223)	(0.0474)	(0.0354)	(0.0270)	(0.0669)

*** indicates significance at the 1% level

** indicates significance at the 5% level, and * indicates significance at the 10% level. Standard errors are reported in parentheses.

### 4.4 Interaction effects of leverage structure with other variables

In this section, we investigate the interaction effects of leverage structure with ROA, STATE and SIZE, where SIZE is defined as the natural logarithm of market capitalization at the end of each year, because we argue that the marginal effect of leverage structure will be affected by these variables. To capture the interaction effects, we add interaction terms in the regression model.

$${SYNCH}_{it}=\lambda_{0}+\lambda_{1}X_{it}+\lambda_{2}X_{it}*Z_{it}+\sum_{k}\lambda_{k}{CONTROL}_{it}^{k}+\varepsilon_{it}$$ (5)

where *X* represents LEV, OPDT and FINDT, respectively. Z denotes ROA, STATE and SIZE, respectively.

Empirical results are presented in Table 7. Econometrically, as interaction terms are included in regressions, then the explanation of the coefficient of leverage structure changes. The marginal effect of *X* now is equal to *λ*_1_+*λ*_2_*Z*, then the coefficient of *X* is equal to *λ*_1_ when *Z* = 0, which doesn’t make any sense in terms of economic explanation. As a result, *λ*_1_ is not our priority. We concern more about the coefficient of interaction terms. From column (1) and (2), we conclude that, the negative effect of LEV (from Table 5) will be attenuated as ROA increases, which means that for firms with higher profitability, the effect of leverage on stock price synchronicity is lower. Generally, when firms use their own earnings to finance a new investment, they choose to borrow money only when earnings are not sufficient, as a result, higher profitability indicates lower leverage. But this interaction effect differs in OPDT and FINDT. This effect remains the same for FINDT but doesn’t exist for OPDT, which means that, the impact of OPDT will not be affected by ROA, and the increase of ROA will reduce FINDT’s ability of incorporating private information. Because FINDT shows higher correlation with firm’s profitability than OPDT. From column (3) and (4), it shows that, when firm is state-owned, it will help LEV to incorporate private information, the same result is found for OPDT and FINDT. In Chinese stock market, the leverage of state-owned firms is more informative for investors, because the public pay more attention to state-owned companies, information of both leverage and its structure will be more valuable for investors. Hence, state-owned firms help to add private information. From column (5) and (6), it shows that, larger market value will help LEV to reveal more private information. But again, the interaction effect differs in OPDT and FINDT. This effect remains the same for FINDT but doesn’t exist for OPDT. Same reasoning as the interaction effect with state-owned firms, the leverage of firms with large market value is more informative, because these kind of firms draw more attention. But unlike state-owned firms, the interaction effect with OPDT and FINDT differs, because FINDT contains more information of firm’s earnings. From the results above, we conclude that, from the perspective of leverage, more state-owned companies and more firms with higher market value will be helpful for the stock market, which help to increase the informativeness of stock market.

**Table 7:** Results of estimation of the interaction effects of leverage structure with ROA, STATE and SIZE. Dependent variable: SYNCH. The individual and time fixed effect are controlled with White-robust standard errors. To save space, the estimation results for other control variables are not reported.

Variables	(1)	(2)	(3)	(4)	(5)	(6)
LEV	-0.335***		-0.149*		1.136**	
	(0.0677)		(0.0823)		(0.568)	
OPDT		-0.258***		-0.0733		-0.415
		(0.0960)		(0.127)		(0.758)
FINDT		-0.372***		-0.195**		2.275***
		(0.0756)		(0.0986)		(0.692)
LEV*ROA	1.921**					
	(0.822)					
OPDT*ROA		0.822				
		(1.208)				
FINDT*ROA		2.543***				
		(0.971)				
LEV*STATE			-0.297***			
			(0.104)			
OPDT*STATE				-0.358**		
				(0.152)		
FINDT*STATE				-0.260**		
				(0.120)		
LEV*SIZE					-0.0958**	
					(0.0380)	
OPDT*SIZE						0.00745
						(0.0508)
FINDT*SIZE						-0.171***
						(0.0459)
ROA	-1.925***	-1.800***	-1.093***	-1.110***	-1.042***	-1.047***
	(0.433)	(0.449)	(0.204)	(0.206)	(0.207)	(0.208)
SIZE					-0.134***	-0.143***
					(0.0267)	(0.0267)
STATE	0.107***	0.107***	0.259***	0.266***	0.111***	0.111***
	(0.0413)	(0.0413)	(0.0693)	(0.0706)	(0.0415)	(0.0416)
Firm FE	Yes	Yes	Yes	Yes	Yes	Yes
Year FE	Yes	Yes	Yes	Yes	Yes	Yes
R-sq	0.474	0.474	0.474	0.474	0.478	0.478

*** indicates significance at the 1% level

** indicates significance at the 5% level, and

* indicates significance at the 10% level. Standard errors are reported in parentheses.

## 5 Robustness check

To check the robustness of the empirical results, one modification is made, we change the data frequency from year to quarter. We investigate whether this relationship is held in short run. The results are reported in Table 8. From the results, the impact of leverage structure is still negative with quarterly data. This finding shows that, in both annual and quarterly data, leverage structure always helps to incorporate private information in stock prices. But the coefficient of leverage structure in quarterly set is lower, which shows that, leverage structure plays a more important role in long run. Moreover, in our model specifications, leverage structure variables are accounting ones, thus, they can be far different from market variables. Follow previous studies [53], we change leverage structure variables by market variables. More specific, for leverage and leverage structure, all variables are divided by market value of equity plus total liabilities instead of being divided by total assets. Empirical results are repsented in Table 9. Our findings are still consistent with accounting variables.

**Table 8:** Results of estimation of the impact of leverage structure on stock price synchronicity with quarterly data. Dependent variable: SYNCH. Two different estimation methods are used. Columns (1) and (2) represent pooled regression with White-robust standard errors; We control the individual and time fixed effect with White-robust standard errors in columns (3), (4) and (5).

	(1)	(2)	(3)	(4)	(5)
LEV	-0.00218***			-0.00384***	
	(0.000346)			(0.000473)	
OPDT		-0.00204***			-0.00349***
		(0.000454)			(0.000663)
FINDT		-0.00229***			-0.00405***
		(0.000399)			(0.000548)
ROA	-3.705***	-3.720***	0.757***	0.253	0.233
	(0.258)	(0.258)	(0.218)	(0.225)	(0.227)
SALE	0.0236***	0.0235***	-0.0245**	-0.00334	-0.00380
	(0.00493)	(0.00495)	(0.0102)	(0.0104)	(0.0104)
STATE	0.183***	0.183***	-0.0467	-0.0427	-0.0424
	(0.0126)	(0.0126)	(0.0476)	(0.0473)	(0.0473)
INST	-0.00389	-0.00314	-0.480***	-0.469***	-0.468***
	(0.0323)	(0.0323)	(0.0332)	(0.0331)	(0.0331)
TURN	6.037***	6.044***	-9.917***	-9.995***	-9.987***
	(0.331)	(0.331)	(0.349)	(0.348)	(0.348)
TOBIN	0.0322***	0.0317***	-0.156***	-0.154***	-0.154***
	(0.00791)	(0.00796)	(0.00879)	(0.00878)	(0.00878)
GDP	-0.00027***	-0.00027***	0.00644***	0.00661***	0.00661***
	(2.49e-05)	(2.49e-05)	(0.00171)	(0.00171)	(0.00171)
SHIBOR	-0.377***	-0.377***	-6.732***	-6.848***	-6.854***
	(0.0106)	(0.0106)	(1.801)	(1.801)	(1.801)
CONS	-1.455***	-1.453***	-3.945***	-4.326***	-4.322***
	(0.105)	(0.105)	(1.114)	(1.112)	(1.112)
Firm FE	No	No	Yes	Yes	Yes
Year FE	No	No	Yes	Yes	Yes
R-sq	0.038	0.038	0.610	0.610	0.610

*** indicates significance at the 1% level

** indicates significance at the 5% level, and * indicates significance at the 10% level. Standard errors are reported in parentheses.

**Table 9:** Market variables of leverage structure. Dependent variable: SYNCH. Two different estimation methods are used. Columns (1) and (2) represent pooled regression with White-robust standard errors; We control the individual and time fixed effect with White-robust standard errors in columns (3) and (4).

	(1)	(2)	(3)	(4)
LEV	-2.449***		-2.244**	
	(0.740)		(0.998)	
OPDT		-2.815***		-2.090**
		(0.772)		(1.013)
FINDT		-2.765***		-2.118**
		(0.767)		(1.004)
ROA	-0.711***	-0.616***	-0.701***	-0.729***
	(0.164)	(0.171)	(0.190)	(0.195)
SALE	0.107***	0.105***	-0.164***	-0.163***
	(0.00737)	(0.00749)	(0.0178)	(0.0179)
STATE	0.236***	0.237***	0.114***	0.113***
	(0.0135)	(0.0135)	(0.0418)	(0.0418)
INST	-0.215***	-0.214***	0.0588	0.0586
	(0.0357)	(0.0357)	(0.0469)	(0.0468)
TURN	-1.661***	-1.642***	-4.472***	-4.481***
	(0.392)	(0.392)	(0.470)	(0.470)
TOBIN	-0.112***	-0.111***	-0.0778***	-0.0781***
	(0.00888)	(0.00893)	(0.0127)	(0.0128)
GDP	-0.383***	-0.385***	-0.567***	-0.566***
	(0.00822)	(0.00828)	(0.0105)	(0.0106)
SHIBOR	-0.406***	-0.406***	0.243***	0.244***
	(0.00764)	(0.00764)	(0.0511)	(0.0511)
CONS	2.663***	3.046***	5.949***	5.782***
	(0.747)	(0.780)	(1.028)	(1.045)
Firm FE	No	No	Yes	Yes
Year FE	No	No	Yes	Yes
R-sq	0.233	0.233	0.477	0.477

*** indicates significance at the 1% level

** indicates significance at the 5% level, and * indicates significance at the 10% level. Standard errors are reported in parentheses.

## 6 Conclusion

Current research shows that there is no consensus for the effect of leverage on stock price synchronicity. This non-consensus may be caused by two reasons. First, the measures of leverage in different contexts differs. The second reason is that the mechanism of the impact of leverage has not been fully investigated. In this paper, we try to provide evidence of these two explanations. For the first one, the measure of leverage used in this paper is the book value of total liabilities divided by total assets. Moreover, we break leverage into two parts: Operating leverage and financing leverage. Operating leverage arises when firms borrow money in running operations from customers and suppliers, and financing leverage occurs when firms borrow money to finance operations from investors [13]. With a sample of the Chinese stock market for the period from 2007 to 2016, our empirical results show, operating and financing leverage have negative impact on stock price synchronicity. Moreover, the impact of total leverage is higher when stock price synchronicity is lower, but on the contrary, the impact of operating leverage and financing leverage is higher when stock price synchronicity is higher. Furthermore, the marginal effect of financing leverage will be attenuated as the profitability of firms becomes higher, but the interaction effect doesn’t exist for operating leverage. On the contrary, the marginal effect of financing leverage will be enhanced as the market capitalization of firms becomes larger, again, it doesn’t exist for operating leverage. Finally, as firms are state-owned, the marginal effect of both operating and financing leverage will be higher.

This paper contributes to current studies of stock price synchronicity in the following aspects. First of all, to better understand the mechanism of the impact of leverage, we break leverage into operating leverage and financing leverage, which has never been done in previous literature. This breakdown reveals the impact of different component of leverage. Moreover, current studies focus on the mean regression model, which only provide average effect of leverage. In this paper, we employ the quantile regression model to investigate the impact of leverage on different level of stock price synchronicity, which provides us a more comprehensive picture. Finally, to investigate the differences across firms, we apply the interaction effect. However, due to the problem of missing data, our sample is unbalanced panel data, which also limits our model selection. For further direction of research, one is to complete the sample by apply nonparametric methods. The second is employing latest econometric techniques to relieve possible endogeneity problems.

## Supporting information

S1 Data (ZIP)
